# Adipose-Derived Stem Cells Alleviate Radiation-Induced Muscular Fibrosis by Suppressing the Expression of TGF-*β*1

**DOI:** 10.1155/2016/5638204

**Published:** 2015-11-16

**Authors:** Wei Sun, Xinchu Ni, Suping Sun, Leiming Cai, Jingping Yu, Jian Wang, Bin Nie, Zhiqiang Sun, Xinye Ni, Xiufeng Cao

**Affiliations:** Department of Radiotherapy, The Affiliated Changzhou No. 2 People's Hospital, Nanjing Medical University, Changzhou 21300, China

## Abstract

We aim to investigate the effects of adipose-derived stem cells (ASCs) transplantation on irradiation-induced skeletal muscle fibrosis. Sixty-four rabbits were randomly divided into ASCs group and PBS group followed by irradiation at unilateral hip with a single dose of 80 Gy. Nonirradiated side with normal skeletal muscle served as normal control. Skeletal muscle tissues were collected from eight rabbits in each group at 1 w, 4 w, 8 w, and 26 w after irradiation. Migration of ASCs was observed in the peripheral tissues along the needle passage in the injured muscle. The proportion of the area of collagen fibers to the total area in sections of ASCs group was lower than those of PBS groups at 4 w, 8 w, and 26 w after irradiation. Significant decrease was noted in the integrated optimal density of the transforming growth factor *β*1 (TGF-*β*1) in the ASCs group compared with those of PBS group at 4 w, 8 w, and 26 w after irradiation. Moreover, the expression of TGF-*β*1 was lower in the ASCs group compared to those of the PBS group at each time point determined by Western blot analysis. ASCs transplantation could alleviate irradiation fibrosis by suppressing the level of TGF-*β*1 in the irradiated skeletal muscle.

## 1. Introduction

Radiotherapy has been commonly used to treat cancer with an aim of increasing the mean life expectancy of patients. Nevertheless, it usually induces normal tissue injury, among which radiation fibrosis is a common complication characterized by excess fibroblast proliferation and collagen fibers deposition [1–4]. To our knowledge, fibrotic lesions after radiotherapy have been reported in a majority of tissues and/or organs including muscles. For example, oral cancer patients experienced different degrees of trismus evoked by radiation-induced injury to masticatory muscles after intensity modulation radiated therapy [5, 6]. In addition, swallowing and voice disorders were induced by the thyroarytenoid injury after radiation [7, 8]. For breast cancer patients, the total radiation field may include the neck, shoulder, axillary, and thoracic muscles. On this occasion, radiation-induced fibrosis is becoming a common and crippling side effect, leading to muscle imbalance with a lessened range of motion, as well as pain and dysfunction [9]. Further, patients undergoing prostate radiotherapy showed defecation urgency and fecal incontinence which was associated with the pelvic floor muscles and anorectal injury after radiation [10].

Currently, adipose-derived stem cells (ASCs) have been highly emphasized on regenerative medicine considering their high proliferation rates and multidirectional differentiation capacity [11]. Under the defined conditions, ASCs can differentiate into multiple lineages and several cell types such as adipose cells, cartilage cells, osteoblasts, and muscle cells [12]. Currently, progress in the understanding of stem cell biology has contributed to the regeneration and/or repair of tissues with radiation injury [13]. Nowadays, to select suitable stem cell type for stimulating tissue regeneration in victims with fibrosis after radiation has been well acknowledged [14]. For instance, ASCs with overexpression of hepatocytes growth factor (HGF) could ameliorate radiation-induced liver fibrosis through downregulating the expression of *α*-SMA and fibronectin [15]. In addition, adipose-derived mesenchymal stem cells showed antifibrotic effects via stimulating the expression of endogenous hepatocytes growth factor (HGF) and prostaglandin E2 [16]. These lead us to investigate the potential roles of ASCs in irradiation-induced skeletal muscle fibrosis.

In this study, ASCs were isolated from New Zealand rabbit and transplanted into the irradiated site to investigate the effect of ASCs implantation on the radiation-induced muscle fibrosis, based on which we provide a therapeutic strategy for ameliorating the irradiated skeletal muscle injury.

## 2. Materials and Methods

### 2.1. Animals and Agents

Healthy New Zealand white rabbits [26 weeks old, weighting 3000 ± 500 g, with an approval number of SCXK (Su) 2007-2008, and a certificate number of 0004282] were purchased from Qinglongshan Animal Breeding Center (Nanjing, China). The animals were raised in standard cages with a temperature of 21 ± 1°C at a relative humidity of 55%. All the animals were kept in 12 h-12 h light-dark cycles and had free access to food and water. The study protocols were in line with the Principles of Laboratory Animal Care formulated by the National Society for Medical Research and the Guide for the Care and Use of Laboratory Animals (NIH publication 85-23, revised in 1996). This study was approved by the Ethical Committee of the Affiliated Changzhou number 2 People's Hospital (Changzhou, China).

Collagenase type I was purchased from Sigma-Aldrich Corporation (St. Louis, MO, USA). Dulbecco's modified eagle medium (DMEM), fetal bovine serum (FBS), and CellTracker CM-DiI were purchased from Life Technologies (Carlsbad, CA, USA). CD90-FITC/IgG-FITC antibodies were purchased from Abcam (Hong Kong) Ltd. (New Territories, HK, China). Rabbit anti-CD31 and rabbit anti-CD34 RBITC conjugated were purchased from Beijing Biosynthesis Biotechnology Co., Ltd. (Beijing, China). EliVision plus kit and DAB kit were purchased from Maixin Biotechnology Development Co., Ltd. (Fuzhou, China). Commercial kits for total protein extraction, SDS-PAGE, ECL, Western blot, and Bradford Protein Assay were obtained from KeyGEN Biological Technology Development Co., Ltd. (Nanjing, China).

### 2.2. Isolation and Culture of ASCs

Subcutaneous fatty tissues were harvested from each rabbit in the back region and/or iliac region according to the previous description [17]. Then the tissues were cut into pieces and digested with collagenase type I (0.1% w/v) at 37°C for 90 min, followed by washing with phosphate buffered saline (PBS) buffer. Afterwards, high-density stromal vascular pellets were obtained after centrifugation at 1,500 rpm for 10 min, followed by resuspending with DMEM medium supplemented with 10% fetal bovine serum. The cell suspension was inoculated at 37°C with 5% CO_2_ in a humidified atmosphere, and the nonadherent cells were removed by washing with PBS 24 hours after the incubation. Finally, the cells were harvested until a confluence of 80%–90% was reached.

### 2.3. Identification, Labelling, and Proliferation of ASCs

Cells of passage 3 (P3) in logarithmic growth phase were resuspended at the density of 1 × 10^6^/mL after digestion. Subsequently, cell surface markers of ASCs were determined using flow cytometry (Becton-Dickinson, Becton, NJ, USA). The primary antibodies were rabbit anti-CD 31, rabbit anti-CD34 RBITC conjugated, and fluorescein isothiocyanate-conjugated mouse monoclonal (FITC.MRC OX-7) to CD90/Thy1(FITC) antibodies. Isotype-matched mouse IgGs served as controls. CellQuest software was used for the result analysis of flow cytometry (Becton-Dickinson, Becton, NJ, USA).

CM-Dil labeling was carried out with strict adhesion to the manufacturer's instructions after digestion and resuspension in PBS. In brief, 2.5 *μ*L CM-Dil (1 *μ*g/*μ*L) was inoculated with the cells (P2) suspension at 37°C for 5 min, followed by suspension at 4°C for 15 min. After that, the mixture was centrifuged at 1,500 rpm for 5 min, and the cells were resuspended using PBS to remove the free CM-Dil. Cells labeled with CM-Dil were cultured under dark conditions.

### 2.4. Experimental Design

Rabbits were anesthetized using 1.5% pentobarbital sodium via intraperitoneal injection. The animals were fixed on a prone position that allowed a 100 cm source skin distance of each buttock (5 cm in diameter) to be sequentially exposed to single fraction external beam irradiation at a dose rate of 300 cGy/min. On this basis, a single dose irradiation (80 Gy) was randomly given to one side of the buttock of each animal using 9 MeV electron rays from a linear accelerator (Primus-Plus, Siemens, Germany). The maximum dose depth was approximately 2 cm.

Twenty-four hours after irradiation, the animals (*n* = 64) were randomly divided into the following: ASCs group (*n* = 32), which was subjected to local intramuscular injection at the center of the irradiation field injection of 5 × 10^7^ ASCs labeled with CM-Dil dissolved in 1 mL PBS, and PBS group (*n* = 32), which was subject to intramuscular injection of 1 mL PBS alone. The body side that underwent no irradiation of each rabbit was set as normal control. Eight animals in each group were randomly sacrificed using air embolism through auricular vein at 1 w, 4 w, 8 w, and 26 w after local irradiation, respectively. Three pieces of muscular tissues (about 2 cm) were obtained from the region at the center of the irradiation field to prepare for frozen section, paraffin section, and Western blot analysis. For the preparation of paraffin section, the sections were fixed using 10% formaldehyde and were embedded in paraffin for further analysis including Masson staining and immunohistochemistry staining.

### 2.5. Masson Staining

The images of Masson staining were observed using an inverted fluorescence microscope IX71-22FL/PH (Olympus Corporation, Tokyo, Japan). Five microscopic fields (200x) of each section were randomly selected. The area of positive collagen fibers and total area of cells were analyzed with Image-Pro Plus 6.0 software (Media Cybernetics, Bethesda, USA), based on which we determine the percentage of collagen fiber in each group.

### 2.6. Immunohistochemistry

Immunohistochemistry was performed to evaluate the expression of TGF-*β*1 in the muscular tissues. Fixed samples were incubated with rabbit anti-TGF-*β*1 at 37°C for 2 h after a series of processing including tissue fixation, dewaxing, hydration, antigen retrieval, and the elimination of endogenous peroxidase. Subsequently, an enhancer (reagent A) and rabbit IgG (Fab fragment) antibody (HRP) (reagent B) were added successively for antibody action at 37°C. Hematoxylin counterstaining was performed after addition of DAB staining solution. All samples were sealed with neutral gum followed by ethanol dehydration. Meanwhile, reactions with PBS replacing the first antibody were performed and served as negative control. A semiquantitative method that judged the immunohistochemistry values as a percentage of positive cells was carried out to determine the expression of TGF-*β*1 [18]. The resulting images were analyzed with Image-Pro Plus 6.0 software. For each section, five microscopic fields (200x) were randomly selected for the assessment of the integrated optimal density (IOD) of TGF-*β*1 positive cells.

### 2.7. Western Blot Analysis

Western blotting analysis was performed as previously described [19]. In brief, muscular tissues (200 mg) were homogenized in RIPA lysis buffer containing protease and phosphatase inhibitors. Proteins were separated by 10% SDS-PAGE and transferred to a Hybond-P PVDF membrane. Subsequently, the membrane was blocked in 5% nonfat milk for 1.5 h and incubated with TGF-*β*1 primary antibody (1 : 200 dilution) overnight at 4°C, followed by incubation with the secondary antibody for 1.5 h at room temperature. The same membrane was probed for *β*-actin for loading control. After washing with Tris-Buffered Saline with Tween 20 (TBST) solution, the proteins were visualized with an ECL kit. The immune complex was quantified by photodetection. The relative density of TGF-*β*1 to *β*-actin was analyzed with the Gel-Pro Analyzer 3.2 (Media Cybernetics, Rockville, MD, USA).

### 2.8. Statistical Analysis

Data analysis was performed using SPSS 16.0 software (SPSS Inc., Chicago, IL, USA). All the data were presented as mean ± standard deviations (SD). Student's *t*-test was carried out to evaluate the intergroup comparison. *P* < 0.05 was considered as statistical difference.

## 3. Results

### 3.1. Morphology of ASCs

**Figure 1:**
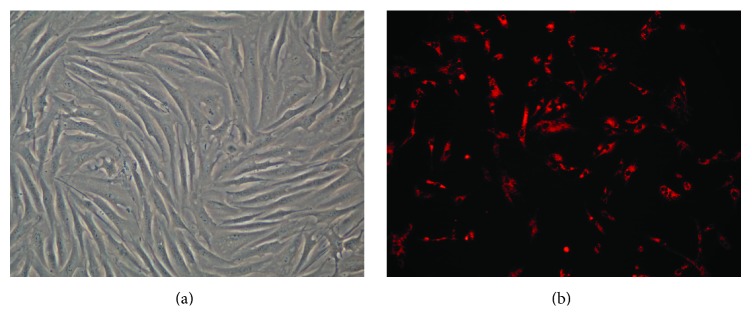
(a) Morphology of ASCs (P2) under an inverted phase contrast microscope; (b) CM-Dil labeled ASCs (P2) under a fluorescence microscope. The images were observed under a magnification of 100x.

The microscopy revealed that fibroblast-like ASCs exhibited long spindle-like shape with clear cellular boundaries. For each cell, the nucleus was well stacked in the center (Figure 1(a)). Cells labeled with CM-Dil showed red membrane and cytoplasm. Morphological evaluation of the cells showed no statistical difference after CM-Dil labeling (Figure 1(b)).

### 3.2. Identification of ASCs Cell Surface Markers

**Figure 2:**
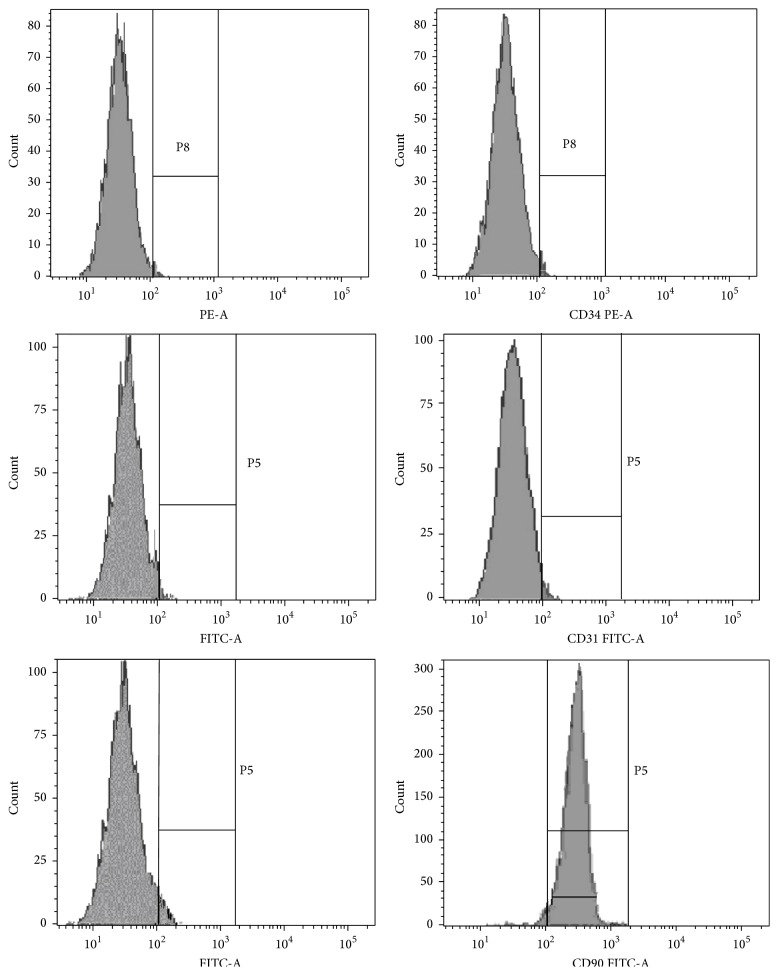
The cell surface markers of cultured ASCs. Flow cytometry indicated that ASCs represented a homogenous population of cells with low expression of CD31 (0.3%) and CD34 (0.2%) and high expression of CD90 (93.1%).

No expression of endothelial marker CD31 or haematopoietic marker CD34 was noticed in ASCs. Most of the ASCs expressed high levels of CD90 adhesion marker (Figure 2).

### 3.3. Migration of ASCs in Muscular Tissues

**Figure 3:**
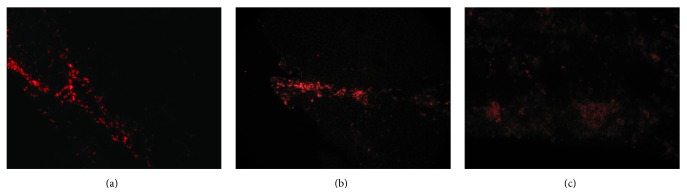
Distribution and migration of CM-Dil labeled ASCs. (a) ASCs distributed along the needle passage at 1 w after implantation; (b) ASCs distributed diffusely in the skeletal muscle tissue at 4 w after implantation; (c) the distribution of ASCs became more diffuse accompanied by the attenuated fluorescence intensity at 8 w after implantation. The images were observed under a magnification of 200x.

No fluorescent color was identified in the muscle tissues in normal control and PBS group, respectively. For the ASCs implantation group, CM-Dil labeled ASCs presented red fluorescence. After transplantation, the cells migrated to the surrounding muscle cells along the needle passage and the fluorescent color was gradually decreased (Figure 3).

### 3.4. ASCs Implantation Inhibited the Formation of Collagen Fiber in Skeletal Muscular Tissues

**Figure 4:**
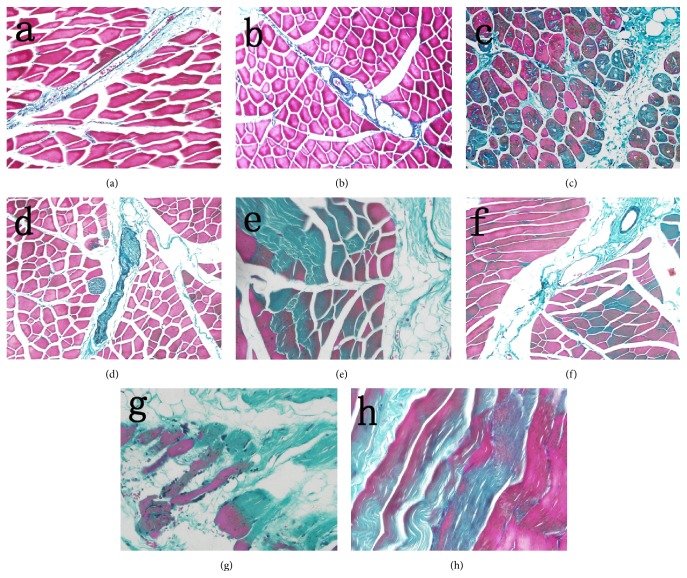
Observation of fibrosis in collagen at different time points after irradiation with Masson staining (×200). Slight swelling of muscle cells was noted in PBS group (a) and ASCs group (b), and little collagen proliferation was observed between muscle cells at 1 w after irradiation; the swelling of muscle cells and collagen proliferation in PBS group (c) were more severe than those of ASCs group (d) at 4 w after irradiation; the denaturation, necrosis of muscle cells, and collagen proliferation in PBS group (e) showed significant increase compared with those in ASCs group (f) at 8 w after irradiation; significant increase of necrotic muscle cells was noted in PBS group (g) in comparison with ASCs group (h) at 26 w after irradiation. Meanwhile, residual skeletal muscle cells were observed in the proliferative collagen in both groups.

**Figure 5:**
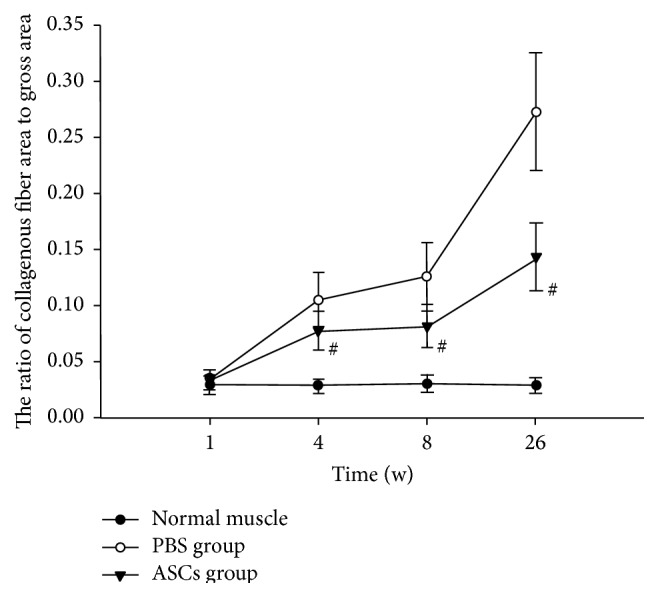
The proportion of collagen fiber in total muscle tissue of each Masson staining image at different time points after irradiation (^#^ *P* < 0.05 versus PBS group).

According to the Masson staining, the cytoplasm of muscle cells was in a red color, while the collagen fiber was in a blue-green color. In normal group, muscle fibers were integrated and arranged in order with no significant cellular atrophy. In addition, a small amount of collagen fibers was observed between the muscle cells, whereas the expression of collagen fiber in ASCs group and PBS group was increased compared with that in the normal group one week after irradiation (Figures 4(a) and 4(b)). With time extension after irradiation, the content of collagen fiber elevated gradually in each group (Figures 4(c)–4(h)). Compared with PBS group, significant decrease was noted in the areas of collagen fiber in total tissue of ASC group at 4 w, 8 w, and 26 w after irradiation (*t* = 4.615, 5.994, and 10.477, *P* < 0.05, Figure 5).

### 3.5. ASCs Implantation Downgraded the Expression of TGF-*β*1

**Figure 6:**
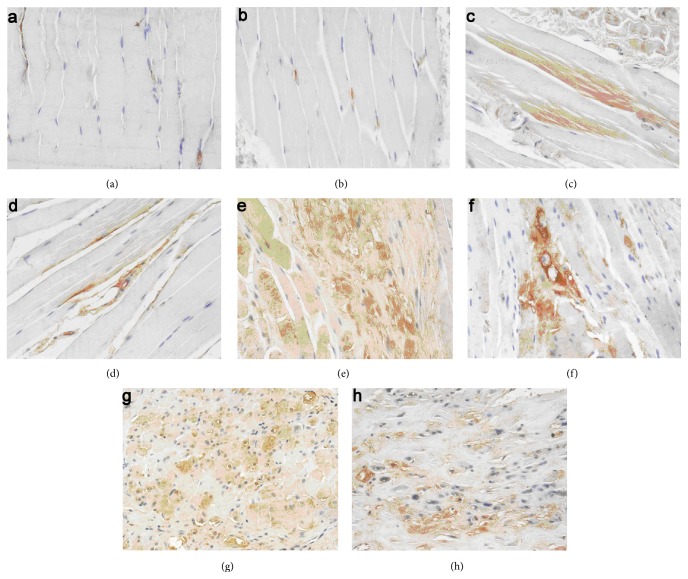
IHC analysis of TGF-*β*1 in muscle tissue at different time points after irradiation (×200). No significant expression of TGF-*β*1 was noted in the PBS group (a) and ASCs group (b) at 1 w after irradiation. The expression of TGF-*β*1 increased gradually in both PBS group (c, e, and g) and ASCs group (d, f, and h) at 4 w, 8 w, and 26 w; however, the expression of TGF-*β*1 in ASCs group was lower than those of PBS group at the same time point after irradiation.

**Figure 7:**
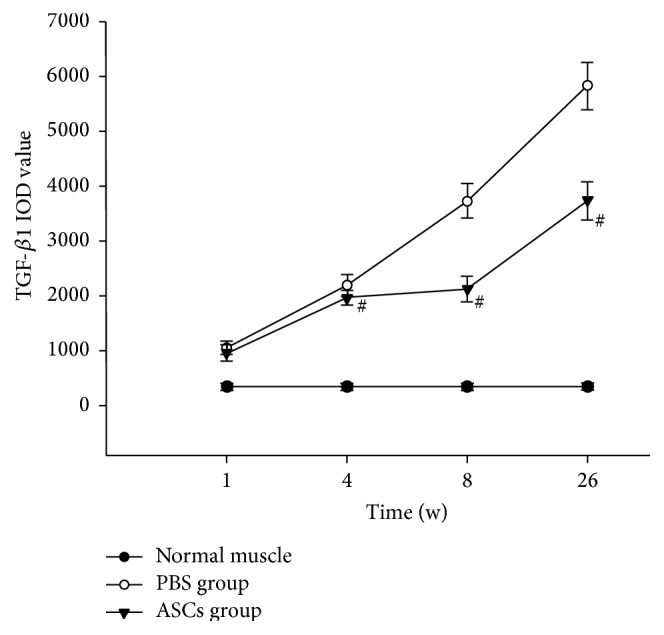
TFG-*β*1 IOD value of each IHC staining image at different time points after irradiation (^#^ *P* < 0.05 versus PBS group).

**Figure 8:**
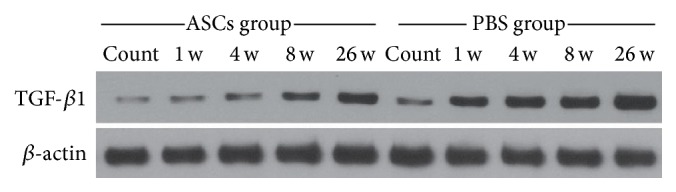
Western blot analysis of the expression of TGF-*β*1 in muscle tissue at different time points after irradiation. Similar results were obtained in three independent experiments. Representative data are shown.

TGF-*β*1 was mainly expressed in the cytoplasm of the muscle cells and was labeled in a brown color. In the normal group, the expression of TGF-*β*1 was low at all time points, and no significant difference was noted in the IOD at each time point. In addition, compared with the normal control group, remarkable increases were revealed in the IOD of TGF-*β*1 in ASCs group and PBS group at 1 w, 4 w, 8 w, and 26 w, respectively (*P* < 0.05). However, significant decrease was noted in the IOD of TGF-*β*1 in the ASCs group compared with those of PBS group at 4 w, 8 w, and 26 w after irradiation (*t* = 3.787, 16.454, and 15.171, *P* < 0.05, Figures 6 and 7). Moreover, Western blot analysis revealed that the expression of TGF-*β*1 was lower in the ASCs group in comparison with those of the PBS group at each time point (*t* = 5.939, 10.249, 5.986, and 7.986, *P* < 0.05, Figure 8).

## 4. Discussion

Skeletal muscle injury induced by high dose irradiation has been commonly reported. In a previous study, Tedla et al. reported a significant reduction in laryngeal intrinsic muscle fibers in patients subject to preoperative radiotherapy with a dose of 60–66 Gy [7]. Persons et al. reported that the hind limb of rats was irradiated with single doses ranging from 15 to 60 Gy. Radiation treatment resulted in muscular wasting, which was more severe at higher doses [20]. Also, Hsu et al. showed that the irradiated gastrocnemius muscle of Wistar rats exhibited a progressive change accompanied by lymphocytic infiltration, hemorrhage, and vascular destruction. Moreover, the amount of collagen between the muscle fibers increased at 12 months after exposing to 80 Gy X-rays [21]. In this study, collagen fibers were gradually increased in skeletal muscles of ASCs group and PBS group with time extension after irradiation of single doses of 80 Gy, which indicated that high dose irradiation could result in skeletal muscle fibrosis.

To date, clinical interventions for irradiation-induced fibrosis mainly include anti-inflammatory, antioxidant, and vascular therapy or hyperbaric oxygen treatment. However, the outcome of such strategies is not satisfactory as the pathological alterations of irradiation injury are considered to be inconvertible [22]. Currently, ASCs therapy represents an alternative option for patients with irradiation injury [13] considering its numerous advantages including abundant stem cell source, low immunogenicity, easy acquisition, and high proliferation capacity [23]. Up till now, it has been well acknowledged that ASCs transplantation has been proved to be effective for the treatment of hepatocirrhosis and idiopathic pulmonary fibrosis [24, 25]. Moreover, ASCs are reported to provide unique opportunities for the functional restoration of the irradiated tissues. For example, Kojima et al. indicated that the transplantation of ASCs into submandibular glands had the potential to restore salivary gland function in mice by improving the saliva flow rate and contributing to the proliferation of blood vessels as well as restoration of the blood flow within submandibular gland tissue after irradiation (10 Gy) [26]. Meanwhile, Akita et al. suggested that autologous ASCs could promote the repair of radiation-induced skin injury [13]. In this study, the severity of fibrosis was more remarkable in both of ASCs group and PBS group after irradiation. Nevertheless, significant decrease was noted in the amount of collagen fibers of ASCs group compared with those of PBS group at each time point after irradiation. This indicated that ASCs implantation at early stage could alleviate muscle fibrosis induced by irradiation.

TGF-*β*1 has been considered as a key modulator for the fibrotic progression involved in the onset and progression of radiation fibrosis [27]. At present, massive studies have demonstrated that fibrosis may be associated with the aberrant production of TGF-*β*1 after irradiation in various tissues such as skin [28], masticatory muscle [29], liver [30], and lung [31]. To be exact, TGF-*β*1 was considered as a vital cause for radiation-induced fibrosis as it was highly expressed in the impaired skin and the underlying muscular fibrotic tissues [28]. Meanwhile, it showed an important role in the initiation of fibrotic cascades in skeletal muscle by downregulating the expression of myogenic proteins and initiating the production of fibrosis-related proteins [32]. Another evidence also indicated that TGF-*β*1 was responsible for the accelerated terminal differentiation of progenitor fibroblasts to functional fibrocytes [33]. In this study, the expression of collagen fibers and TGF-*β*1 in muscle tissue was elevated after irradiation, which indicated that the abnormal expression of TGF-*β*1 may be related to the skeletal muscle fibrosis after high dose irradiation. Besides, IHC and Western blot analysis were performed to evaluate the expression of TGF-*β*1 in the muscular tissues, which indicated significant decrease in the expression of TGF-*β*1 in the ASCs group compared with those of PBS group at 4 w, 8 w, and 26 w after irradiation. On this basis, we concluded that ASCs transplantation could inhibit TGF-*β*1 expression in the irradiated muscle tissue and may further contribute to the alleviation of radiation fibrosis in skeletal muscle. However, the exact mechanism of how ASCs interfere with TGF-*β*1 expression is still not unknown. Besides, it is hard to define whether the modulation of TGF-*β*1 expression could affect the fibrosis extent. According to our published and unpublished data, we hypothesize that ASCs may modulate the protein expression involved in TGF beta-1 signaling pathway. In the future, knockout of gene encoding the protein involved in TGF beta-1 signaling pathway will be undertaken, and we hope that statistical difference may be observed in the irradiation-induced skeletal muscle fibrosis.

In conclusion, ASCs transplantation could alleviate the radiation-induced skeletal muscle fibrosis, which may be associated with the downregulation of TGF-*β*1 in the irradiated muscle tissue. However, further studies are required to investigate the definite mechanism in the role of ASCs-mediated TGF-*β*1 in radiation injury.
